# In vitro promoter recognition by the catalytic subunit of plant phage-type RNA polymerases

**DOI:** 10.1007/s11103-016-0518-z

**Published:** 2016-08-06

**Authors:** Alexandra-Viola Bohne, Marlene Teubner, Karsten Liere, Andreas Weihe, Thomas Börner

**Affiliations:** 1 Institute of Biology, Humboldt University, Philippstr.13, Rhoda Erdmann Haus, 10115 Berlin, Germany; 2 Molecular Plant Sciences, Ludwig-Maximillians-University, Grosshaderner Str. 2–4, 82152 Planegg-Martinsried, Germany; 3 SMB Services in Molecular Biology GmbH, Rudolf-Breitscheidstr. 70, 15562 Rüdersdorf, Germany

**Keywords:** Arabidopsis, Chloroplast RNA polymerase, Mitochondrial RNA polymerase, Phage-type RNA polymerase, Promoter recognition

## Abstract

**Key message:**

We identified sequence motifs, which enhance or reduce the ability of the Arabidopsis phage-type RNA polymerases RPOTm (mitochondrial RNAP), RPOTp (plastidial RNAP), and RPOTmp (active in both organelles) to recognize their promoters in vitro with help of a ‘specificity loop’. The importance of this data for the evolution and function of the organellar RNA polymerases is discussed.

**Abstract:**

The single-subunit RNA polymerase (RNAP) of bacteriophage T7 is able to perform all steps of transcription without additional transcription factors. Dicotyledonous plants possess three phage-type RNAPs, RPOTm—the mitochondrial RNAP, RPOTp—the plastidial RNAP, and RPOTmp—an RNAP active in both organelles. RPOTm and RPOTp, like the T7 polymerase, are able to recognize promoters, while RPOTmp displays no significant promoter specificity in vitro. To find out which promoter motifs are crucial for recognition by the polymerases we performed in vitro transcription assays with recombinant *Arabidopsis* RPOTm and RPOTp enzymes. By comparing different truncated and mutagenized promoter constructs, we observed the same minimal promoter sequence supposed to be needed in vivo for transcription initiation. Moreover, we identified elements of core and flanking sequences, which are of critical importance for promoter recognition and activity in vitro. We further intended to reveal why RPOTmp does not efficiently recognize promoters in vitro and if promoter recognition is based on a structurally defined specificity loop of the plant enzymes as described for the yeast and T7 RNAPs. Interestingly, the exchange of only three amino acids within the putative specificity loop of RPOTmp enabled the enzyme for specific promoter transcription in vitro. Thus, also in plant phage-type RNAPs the specificity loop is engaged in promoter recognition. The results are discussed with respect to their relevance for transcription in organello and to the evolution of RPOT enzymes including the divergence of their functions.

**Electronic supplementary material:**

The online version of this article (doi:10.1007/s11103-016-0518-z) contains supplementary material, which is available to authorized users.

## Introduction

Mitochondria and plastids possess their own genomes and transcription machineries. Both organelles have changed their transcriptional apparatus as compared to their bacterial ancestors, since they are using RNAPs related to the single-subunit RNAPs of bacteriophages like T3 and T7. The T7 RNAP is able to perform all steps of transcription from promoter recognition to termination without additional transcription factors (Sousa and Mukherjee 2003). However, transcription without auxiliary factors is not an inherent feature of all bacteriophage RNAPs. The RNAP of another *Escherichia coli* phage, N4, needs for efficient transcription the *E. coli* single-stranded DNA binding protein (SSB). SSB presents the hairpin-form promoter to the N4 RNAP for binding and participates in the elongation of transcription by separating the DNA/RNA hybrid from the RNAP elongation complex to enable multiple rounds of transcription (Amunts and Nelson 2008). The *Pseudomonas aeruginosa* phage phiKZ and related giant phages encode and use proteins distantly related to the largest subunits of the multisubunit RNAPs of bacteria and eukaryotes (Hansson et al. 2007). In contrast to the T7 RNAP, yeast and mammalian RNAPs of the T3/T7 type (RPOTs) require two auxiliary factors, mtTFA and mtTFB, for accurate and efficient transcription initiation in vivo (Jang and Jaehning 1991; Lisowsky and Michaelis 1988; Schinkel et al. 1987; Shadel and Clayton 1995). mtTFAs belong to the family of HMG-box proteins. Mitochondrial mtTFBs are related to a family of rRNA methyltransferases which dimethylate two adenosines near the 3′ end of the rRNA in the small ribosomal subunit (Park et al. 2009; Richter et al. 2010; Schubot et al. 2001). mtTFB forms a holoenzyme with the core polymerase prior to DNA binding and promoter recognition (Mangus et al. 1994).

The yeast mitochondrial RNAP, RPO41, is capable of initiating transcription without accessory factors in in vitro assays, if the promoter is contained in a supercoiled DNA molecule or experimentally modified to form a bubble around the initiation site to facilitate melting (Matsunaga and Jaehning 2004). Specific promoter recognition by the T7 RNAP as well as the yeast RPO41 enzyme relies on a specificity loop located in the C-terminus of the enzyme and involved in formation of hydrogen bonds between the amino acids of the specificity loop and certain nucleotides of the promoter sequences (Cheetham et al. 1999; Nayak et al. 2009; Rong et al. 1998). Although the specificity loop is conserved in phage-type RNAPs rather structurally than by amino acid sequence, a variable, short sequence was identified that is speculated to be responsible for promoter recognition in most, if not all phage-type RNAPs (Nayak et al. 2009).

Like in most other eukaryotes, mitochondrial transcription in plants solely relies on phage-type RNAPs. In the model plant *Arabidopsis* and all other eudicotyledonous plants studied so far, a small family of three *RPOT* genes codes for such phage-type RNAPs. Aside from *RPOTm* encoding a mitochondrial RNAP, a second RPOT enzyme (RPOTp) is imported into plastids. The third *RPOT* gene (*RPOTmp*) encodes a protein that is dually targeted into both mitochondria and plastids. Consequently, dicotyledonous angiosperms like *Arabidopsis* possess two mitochondrial (RPOTm, RPOTmp) and two plastidial (RPOTp, RPOTmp) phage-type RNAPs. The dual-targeted enzyme is not found in monocotyledonous plants and the basic angiosperm, *Nuphar advena* (Weihe et al. 2012). In addition to phage-type RNAPs (dubbed NEP, from *n*uclear-gene *e*ncoded plastid RNA *p*olymerase), plastids use a *p*lastid-*e*ncoded plastid RNA *p*olymerase (PEP). PEP is not a single-subunit enzyme but related to the bacterial RNAPs. It is composed of four core subunits (α, β, β’ and β′′) and one of several nuclear-encoded sigma factors. In photosynthetically active plastids, the chloroplasts, several additional protein factors are associated with the holoenzyme and essential for transcription (Börner et al. 2015; Pfalz and Pfannschmidt 2013).

Since PEP has evolved from the cyanobacterial RNAP (Yagi and Shiina 2014), this bacterial-type enzyme recognizes σ70-like promoters with conserved −10 (TATAAT) and −35 (TTGACA) elements. NEP recognizes promoter sequences unique to this RNAP. Two structurally different classes of plastidial promoters engaged in transcription by NEP have been dissected in the plastidial genome. Whereas many NEP promoters are characterized by a conserved YRTA motif upstream of the site of transcription initiation, a second class of NEP promoters possess neither the YRTA sequence nor other consensus motifs (for review see Liere et al. 2011). Mitochondrial promoters in angiosperms often contain a consensus CRTA motif, similar to the YRTA motif of plastidial NEP promoters and function also in plastidial transcription (Bohne et al. 2007). In dicotyledonous plants, the CRTA motif is part of a nonanucleotide sequence overlapping the initiation site (Binder et al. 1996; Kühn et al. 2005). However, similar to the situation in plastids, many mitochondrial promoters consist of non-consensus sequences lacking common sequence motifs (Kühn et al. 2005; Zhelyazkova et al. 2012). Promoters of plant phage-type RNAPs differ in their strength (e.g. Bohne et al. 2007), but it has not been investigated yet to which extent certain sequence motifs determine their activity. Moreover, no transcription factor has been identified so far that can support promoter recognition by plant phage-type RNAPs. Neither mtTFA nor mtTFB homologs have been found in plants (Richter et al. 2010). It remains therefore unclear how a large part of plastidial NEP and mitochondrial promoter sequences determine the site of transcription initiation and how plant organellar promoters are recognized by phage-type RNAPs.

Like the yeast enzyme, *Arabidopsis* phage-type polymerases have been shown to utilize a number of organellar promoters, initiate transcription and perform elongation of the transcript without additional co-factors in vitro on supercoiled, but not linear DNA (Kühn et al. 2007). An in vitro transcription assay was used in which recognition of a multitude of mitochondrial promoters and of several plastidial NEP promoters was studied using recombinant *Arabidopsis* RPOTp, RPOTm and RPOTmp polymerases. RPOTp and RPOTm were shown to recognize most of the mitochondrial promoters and one plastidial NEP promoter in vitro. In contrast, the in vitro assays did not reveal a significant ability of RPOTmp to recognize mitochondrial or plastidial promoters (Kühn et al. 2007), although there is evidence for this enzyme to play roles in the transcription of genes in both organelles (Courtois et al. 2007; Kühn et al. 2009; Swiatecka-Hagenbruch et al. 2008). With RPOTm and RPOTp recognizing only part of the investigated promoters and RPOTmp displaying no significant promoter specificity but high non-specific transcription activity in vitro (Kühn et al. 2007), it is evident that the *Arabidopsis* enzymes need auxiliary factors for transcription in organello.

In the present study, we employed in vitro transcription assays with modified promoters, wild-type RPOT enzymes, and an RPOTmp with altered amino acid sequence in the putative specificity loop. Our objectives were to find out to which extent certain promoter motifs are crucial for recognition by the polymerases and how they determine promoter strength, why RPOTmp does not efficiently recognize promoters in vitro, and if the interaction of the *Arabidopsis* enzymes with their promoters is based on a structurally defined specificity loop as known from the T7 polymerase and thus indicating this process to be evolutionary conserved from phages to plants.

## Material and methods

### Preparation of in vitro transcription templates

**Fig. 1:**
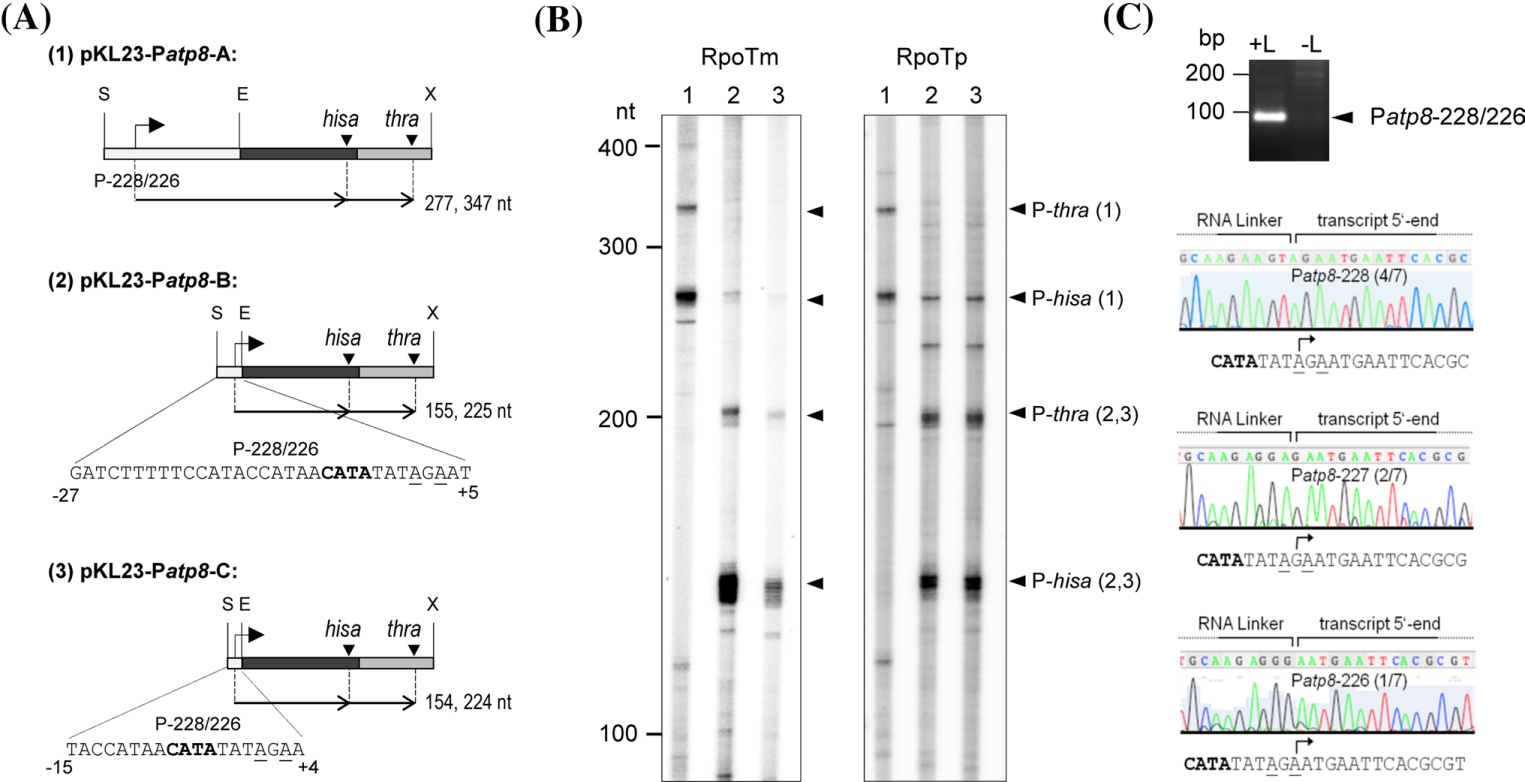
Deletion analysis of the mitochondrial P*atp8*-228/226 promoter. **a** Scheme of vectors used for in vitro transcription of the inserted P*atp8*-228/226 promoter sequences. The vectors contain ~200 bp (1, pKL23-P*atp8*-A), sequences from −27 to +5 (2, pKL23-P*atp8*-B) or −15 to +4 (3, pKL23-P*atp8*-C) around the in vivo transcription initiation sites at −228/226 determined previously by Kühn et al. (2005). For simplification, the scarcely detectable promoter P*atp8*-157 on vector pKL23-P*atp8*-A is not shown. Promoter cores are *written bold*, transcription initiation sites are *underlined*. Transcripts expected from initiation at the P*atp8*-228/226 and termination at *hisa* or *thra* (*arrowheads*) are indicated by *horizontal black arrows* labelled with the respective RNA lengths. **b** Recombinant RPOTm and RPOTp were assayed for promoter-specific transcription from supercoiled vectors (1–3) shown in (**a**). ^32^P-labeled RNA products were separated in 5 % sequencing gels alongside an RNA size marker; sizes are given in nucleotides (marker lane not displayed). Specific RNA products are indicated at the *right* and labelled with the corresponding vector number. Additional minor signals have been observed before and may be due to differently migrating major products (Kühn et al. 2007). **c** 5′-RACE was performed on RNAs synthesized from pKL23-P*atp8*-B by RPOTm as described by Kühn et al. (2009). Transcripts were 5′ ligated to an RNA linker (+L) and subjected to RT-PCR. Non-ligated transcripts served as control (−L). Resulting PCR products were separated on an agarose gel alongside a size marker; sizes are given in base pairs (marker lane not displayed). The specific product corresponding to transcript 5′ ends mapping to P*atp8*-228/226 (*arrowhead*) was subjected to sequencing. The resulting chromatograms demonstrating the RNA linker and ligated transcript 5′ ends are shown below. Determined in vitro transcription initiation sites are indicated by *bent arrows*. Number of sequenced clones as well as frequency of initiation at the respective nucleotide are given within the chromatogram

For the construction of mitochondrial or plastidial DNA templates used for in vitro transcription assays sequences were either PCR-amplified from total *Arabidopsis* DNA or obtained by annealing of complementary primers listed in Supplementary Tables S1 and S2. Products were ligated into *Sac*I/*EcoR*I-cleaved pKL23 (Liere and Maliga 1999a) upstream of two bacterial terminators as described by Kühn et al. (2007; compare Fig. 1a).

To analyse the efficiency of annealing 1 µg of annealing products was separated on native 15 % polyacrylamide gels.

### In vitro transcription assays and 5′ end mapping of in vitro–synthesized RNAs

Specific and unspecific in vitro transcription assays were performed as described previously by Kühn et al. (2007). To allow comparability, the experiments were performed in parallel with equal amounts of identical preparations of recombinant RNA polymerases (400 fmol) and DNA templates (200 ng in a final volume of 15 µL). For quantification of transcript abundances, the background signal of each lane was subtracted from signals corresponding to specific transcripts. 5′ ends of in vitro-synthesized transcripts were determined employing a 5′-RACE technique combined with tobacco acid pyrophosphatase (TAP) treatment as described by Bohne et al. (2007).

### Expression and purification of RNAPs

Recombinant thioredoxin-hexahistidine–tagged RPOTm, RPOTmp, and RPOTp investigated in in vitro transcription assays were expressed and purified under native conditions as described previously by Kühn et al. (2007). For mutagenesis experiments the wild-type sequence of RPOTmp encoding amino acids 107–1011 (locus tag At5g15700), was PCR-amplified with primers listed in Supplementary Table S3 and cloned via *Eco*RI/*Pst*I*- or Sac*I/*Xba*I restriction sites into the pCOLD DNAI vector (TaKaRa Bio Europe S.A.S., Saint-Germain-en-Laye, France) to result in construct pCold-His-RPOTmp. For mutagenesis, RPOTmp point mutations were introduced by site-directed mutagenesis using the Phusion^TM^ Site-Directed Mutagenesis Kit (FINNZYMES, Thermo Fisher Scientific Biosciences GmbH, St. Leon-Rot, Germany) according to the manufacturer’s instructions. Used primers are listed in Supplementary Table S3. Recombinant hexahistidine-tagged enzymes were overexpressed in *E. coli* BL21 Codon Plus RIL (Agilent Technologies, Waldbronn, Germany) from resulting construct pCold-His-RPOTmp (RHR) in parallel with the wild-type enzymes. Protein expression was induced with a final concentration of 1 mM IPTG for 20–24 h at 15 °C. The purification of histidine-tagged proteins over Ni^2+^-NTA agarose was performed as described previously (Kühn et al. 2007).

## Results

### A promoter sequence from −15 to +4 is sufficient for recognition by RPOTm and RPOTp in vitro

Our previous studies revealed in vitro recognition of diverse organellar promoter sequences by the *Arabidopsis* phage-type RNAPs RPOTm and RPOTp (Kühn et al. 2007). However, as the investigated promoter constructs usually contained promoter regions of ~200–300 bp an exact assignment of nucleotides required for promoter recognition was not warranted. We therefore sought to further elucidate the role of certain promoter elements stimulating the transcription by RPOTm and RPOTp.

To narrow down the promoter region required for transcription initiation, DNA templates were constructed by inserting truncated sequences of the mitochondrial promoter P*atp8-228*/*226* (the numbers indicate the position of the in vivo initiating nucleotides with respect to the start of the coding sequence of the gene) into pKL23 upstream of the two bacterial terminator sequences *hisa* and *thra*, which efficiently stop transcription by both RNAPs (Liere and Maliga 1999a; Kühn et al. 2007). The long version of P*atp8-228*/*226* was well recognized in vitro by RPOTm and RPOTp but not by RPOTmp in the previous study (Kühn et al. 2007). The resulting promoter deletion derivatives contained sequences from −27 to +5 (pKL23-P*atp8*-B) or −15 to +4 (pKL23-P*atp8*-C), respectively, and were tested for in vitro transcription by recombinant RPOTm and RPOTp (Fig. 1). Similar to the control vector, which includes ~200 bp around the transcription initiation site, both deletion derivatives were efficiently transcribed by RPOTm and RPOTp indicating that sequences from −15 to +4 are sufficient for promoter recognition (Fig. 1b). However, transcription initiation from pKL23-P*atp8*-C by RPOTm, but not RPOTp, was reduced in comparison to transcription from pKL23-P*atp8*-B, indicating a certain relevance of nucleotides between positions −15 and −27 or at +5 for efficient transcription by RPOTm.

Signals obtained of P*atp8*-228/226-*hisa* transcripts from pKL23-P*atp8*-B and -C with expected sizes of 154 or 155 nt, respectively, appeared as multiple bands (Fig. 1b, lanes 2, 3). To assess whether these bands are due to inaccuracies resulting from less precise initiation at −228/−226 from the truncated promoters, 5′-ends of transcripts initiated at pKL23-P*atp8*-B by RPOTm were determined by 5′-RACE (Fig. 1c). Results showed that major transcript 5′ ends start at one out of two adenines at positions −228/−226 as determined in vivo. Approximately 30 % of analysed transcript 5′ ends started at nucleotide position −227, which indicates only a slight inaccuracy in vitro. Therefore, the occurrence of multiple P-*hisa*- signals might rather be caused by termination at several nucleotides at the AT-rich *hisa* terminator region or potentially formed secondary structures at the transcript 3′ end.

**Fig. 2:**
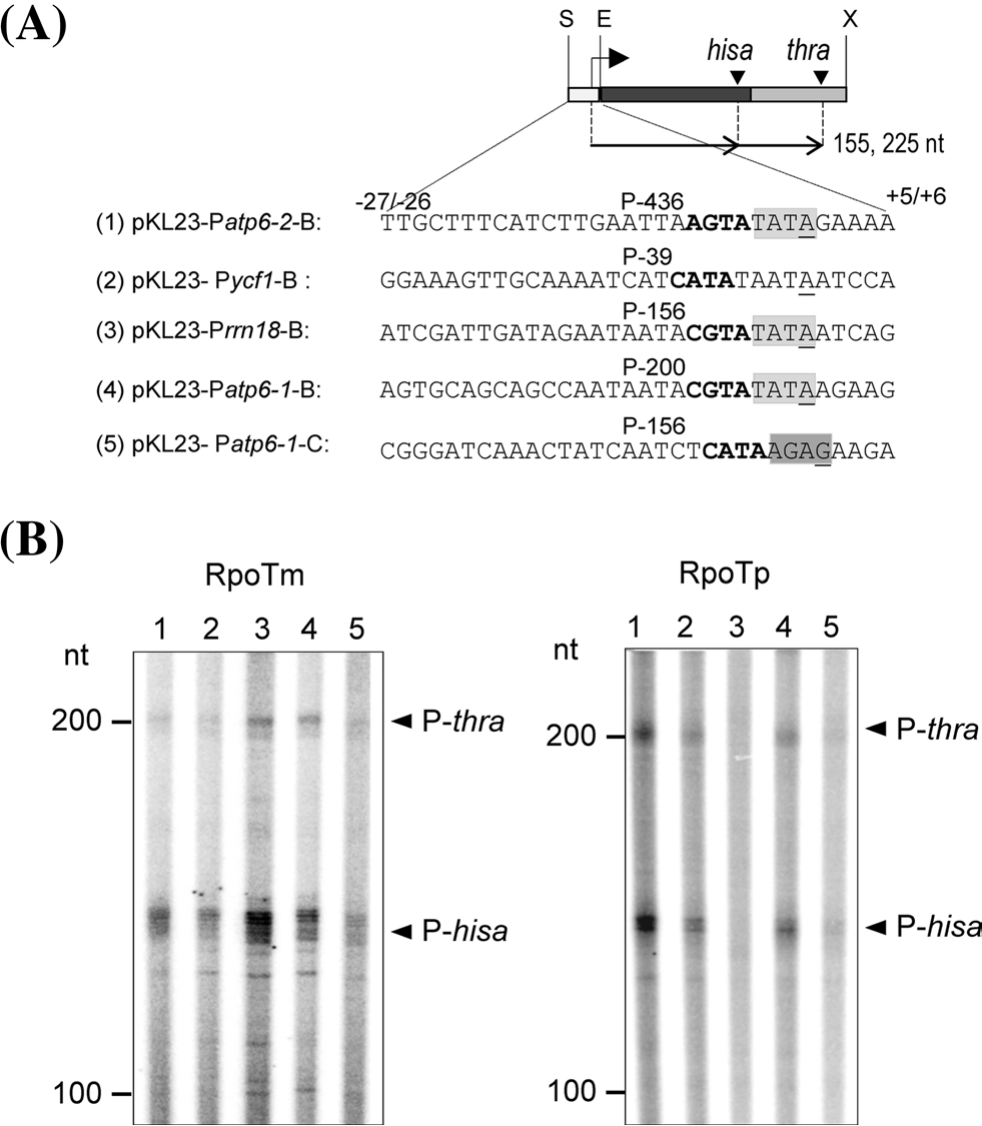
The promoter region of −27 to +6 is sufficient for specific transcription by RPOTm and RPOTp. **a** For construction of pKL23-P*atp6-2*-B 1 pKL23-P*ycf1*-B 2 pKL23-P*rrn18*-B 3 pKL23-P*atp6-1*-B 4, and pKL23-P*atp6-1*-C 5 indicated promoter sequences from −26 to +6 (or −27 to +5 in case of construct five) overlapping the transcription initiation site were inserted into pKL23. Transcripts expected from initiation at the introduced promoters followed by termination at *hisa* and *thra* are indicated. *Symbols* are as in Fig. 1. The motifs TATA and AGAG which are recurring in many mitochondrial and some plastidial NEP promoters, are highlighted in *light* and *dark grey*, respectively. **b** RPOTm and RPOTp were tested for promoter-specific transcription from supercoiled vectors (1–5) depicted in **a**). Transcript analysis and *symbols* are as in Fig. 1

The analysed P*atp8*−228/−226 promoter contains a typical CRTA/YRTA core motif followed by TATA which is seen in many plant organellar promoters recognized by phage-type RNAPs (reviewed in Liere et al. 2011). Nonetheless, a number of identified promoter sequences exhibit alternative architectures, which allow transcription by RPOTm and RPOTp in vitro when ~200–300 bp around the transcription initiation site are provided (Kühn et al. 2005, 2007; Swiatecka-Hagenbruch et al. 2007). Frequently occurring elements within these promoters are an AGTA core sequence as well as an AGAG motif downstream of the promoter core (Fig. 2a). To test if truncated sequences are also sufficient to promote transcription initiation from these alternative promoters, respective regions of the mitochondrial P*atp*6-2-436, P*rrn18*-156, P*atp6-1*-200, P*atp6-1*-156, and the plastidial P*ycf1*-39 were inserted into pKL23 (Fig. 2a) and tested for in vitro transcription by RPOTm and RPOTp. As shown in Fig. 2b, nearly all provided sequences allowed transcription by RPOTm and RPOTp. The only exception was the missing transcription of P*rrn18*-156 by RPOTp which, however, was already only weakly recognized by the enzyme when larger promoter regions were provided (Kühn et al. 2007). Interestingly, the transcription of the P*atp6-1*-156 promoter had not been detected when the vector construct additionally contained the further upstream located promoter P*atp6-1*-200 (Kühn et al. 2007), which implies a competitive usage of both sequences by the RNAPs.

As sequences from −27 to +6 proved to be sufficient for transcription initiation in vitro, appropriate promoter regions were inserted into pKL23 for all following experiments if not indicated otherwise.

### Importance of promoter core motifs for efficient in vitro transcription by RPOTm and RPOTp

**Fig. 3:**
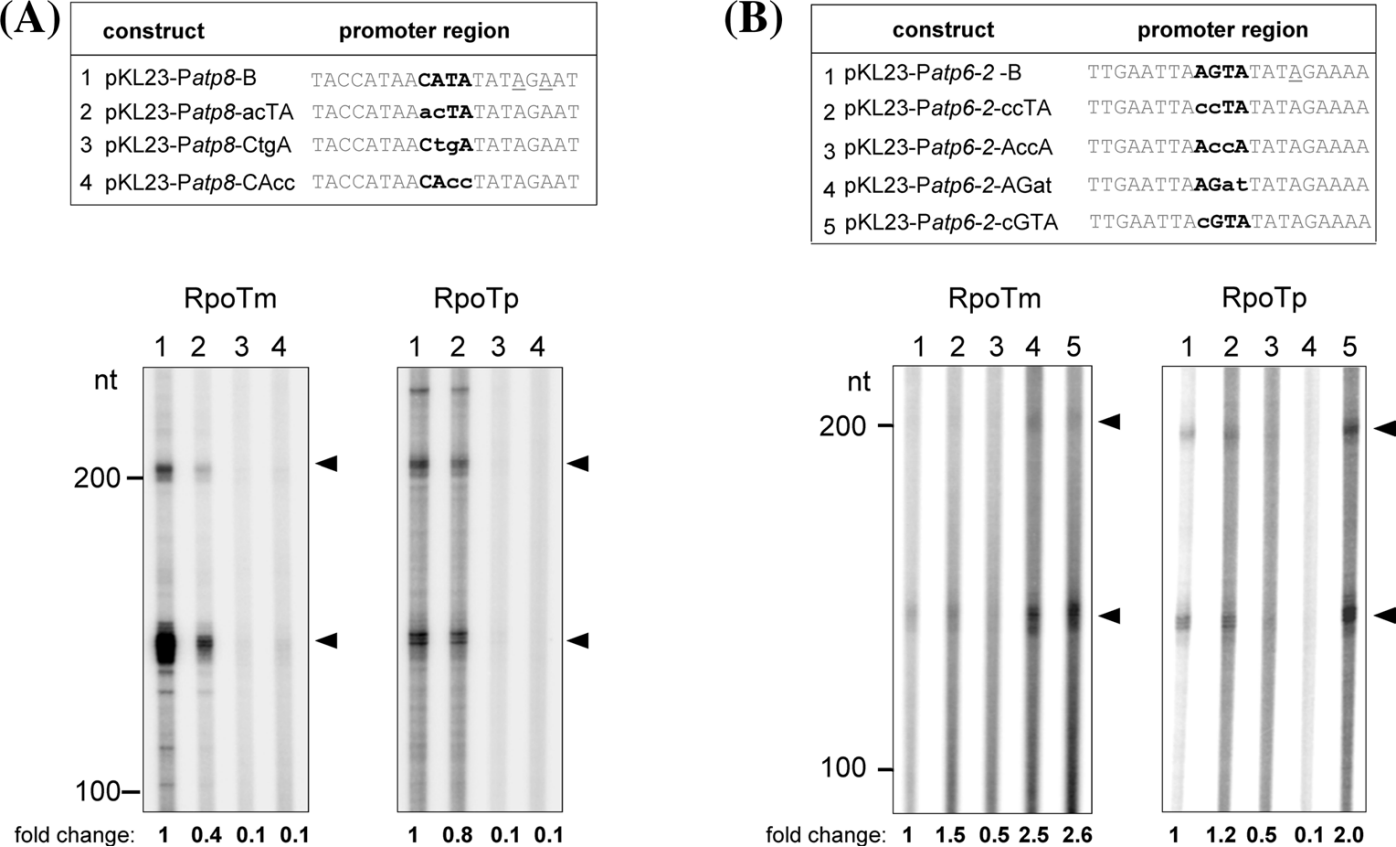
In vitro transcription from mutagenized promoter core motifs. Recombinant RPOTm and RPOTp were assayed for promoter-specific transcription of the mutagenized mitochondrial P*atp8*-228/226 (**a**) and P*atp6-2*-436 (**b**) promoters. *Upper panels* show wild-type and mutagenized promoter sequences which were inserted into pKL23 (compare Figs. 1a, 2b). Nucleotides in *boldface highlight* the promoter core motif targeted by the mutagenesis. The transcription initiation sites are underlined in the wild-type sequence. Transcripts expected from initiation at the introduced promoter followed by termination at *hisa* and *thra* have a length of 155 and 225 nt, respectively. *Lane numbers* in the *lower panels* correspond to promoter sequences shown above. Transcript analysis and *symbols* are as described in Fig. 1. Relative-fold changes for specific transcripts given below the autoradiogram were determined from the mean of two independent experiments

An indication for the importance of the CRTA core motif within the *Arabidopsis* P*atp8*−228/−226 promoter for efficient in vitro transcription has been obtained previously. The substitution of the CATA motif by the sequence CtgA affected the in vitro promoter recognition by RPOTp and RPOTm negatively (Kühn et al. 2007). To provide further evidence for the importance of the YRTA motif, a more detailed analysis of mutagenized promoters was performed. For this purpose, we choose the P*atp8*-228/-226 promoter exhibiting a CATA core motif as well as P*atp6-2*-436 with an AGTA core sequence (Fig. 3). We substituted the CATA motif of the P*atp8*-228/-226 promoter with the sequences acTA, CtgA, or CAcc, respectively, and tested the resulting constructs for transcription by RPOTm and RPOTp (Fig. 3a). The exchange of the first two nucleotides of the CATA motif (acTA) still allowed transcription by both RNAPs. However, specific transcripts synthesized by RPOTm were reduced to ~40 % in comparison to transcripts initiated at the wild-type promoter, while RPOTp revealed a slight reduction of ~20 %. By contrast, an exchange of the two central nucleotides (CtgA) or the last two (CAcc), which destroys the highly conserved TA of the tetranucleotide, led to drastically reduced levels of correctly initiated transcripts for both enzymes.

To learn if mutagenesis of an alternative core sequence similarly affects transcription in vitro, we exchanged the AGTA of P*atp6-2*-436 to ccTA, AccA, or AGat, respectively (Fig. 3b). Additionally, an exchange of only the first nucleotide (cGTA), created a typical CRTA/YRTA motif. Unlike the reduced transcription of the mutagenized CRTA/YRTA motif described above, we observed a reduced initiation only for the template with the modified motif AccA, but a slightly (ccTA) or even considerably (AGat, cGTA) increased transcription by RPOTm from the other mutagenized DNA templates. This effect was less obvious when initiation by RPOTp was analysed. Different preferences of RPOTm and RPOTp for certain nucleotides in the promoter core were clearly observed for the AGat construct, which was transcribed with increased efficiency by RPOTm but hardly recognized by RPOTp. The most pronounced positive effect was seen with RPOTm and RPOTp when a typical CRTA/YRTA motif was generated thus supporting the importance of these residues for promoter recognition by RPOTm and RPOTp. Taken together, the in contrast to the CRTA/YRTA motif only slightly altered transcription of the mutagenized AGTA motif suggests that this core sequence is less important for recognition by the RNAPs. Here, nucleotides flanking the core motif may additionally support promoter recognition.

### Additional promoter elements influence transcriptional activities

The above-described mutagenesis of the promoter core sequences of P*atp8*-228/226 and P*atp6-2*-436 supports the importance of a highly conserved CRTA/YRTA motif to guarantee efficient transcription in vitro. However, high transcriptional rates by RPOTm and RPOTp cannot be achieved by this motif alone, as several promoters carrying a CRTA/YRTA motif, like the mitochondrial P*atp9*-239 or many plastidial NEP promoters, are not transcribed by the RNAPs in vitro. Vice versa, several promoters with alternative promoter cores (e.g. ATTA, AGTA), are transcribed by RPOTm and RPOTp (Kühn et al. 2007). Therefore, additional promoter elements are likely required for the specific and efficient transcription by RPOTm and RPOTp but have not been investigated so far. A further potentially supporting element is the often seen TATA motif downstream of the promoter core, with the last nucleotide representing the initiation site in vivo and in vitro (Kühn et al. 2005, 2007). Another relatively frequent motif at this position is AGAG, with the last G as site of transcription initiation (Kühn et al. 2005). Promoters with AGAG were less efficiently recognized in vitro compared to promoters with the TATA motif (Kühn et al. 2007).

**Fig. 4:**
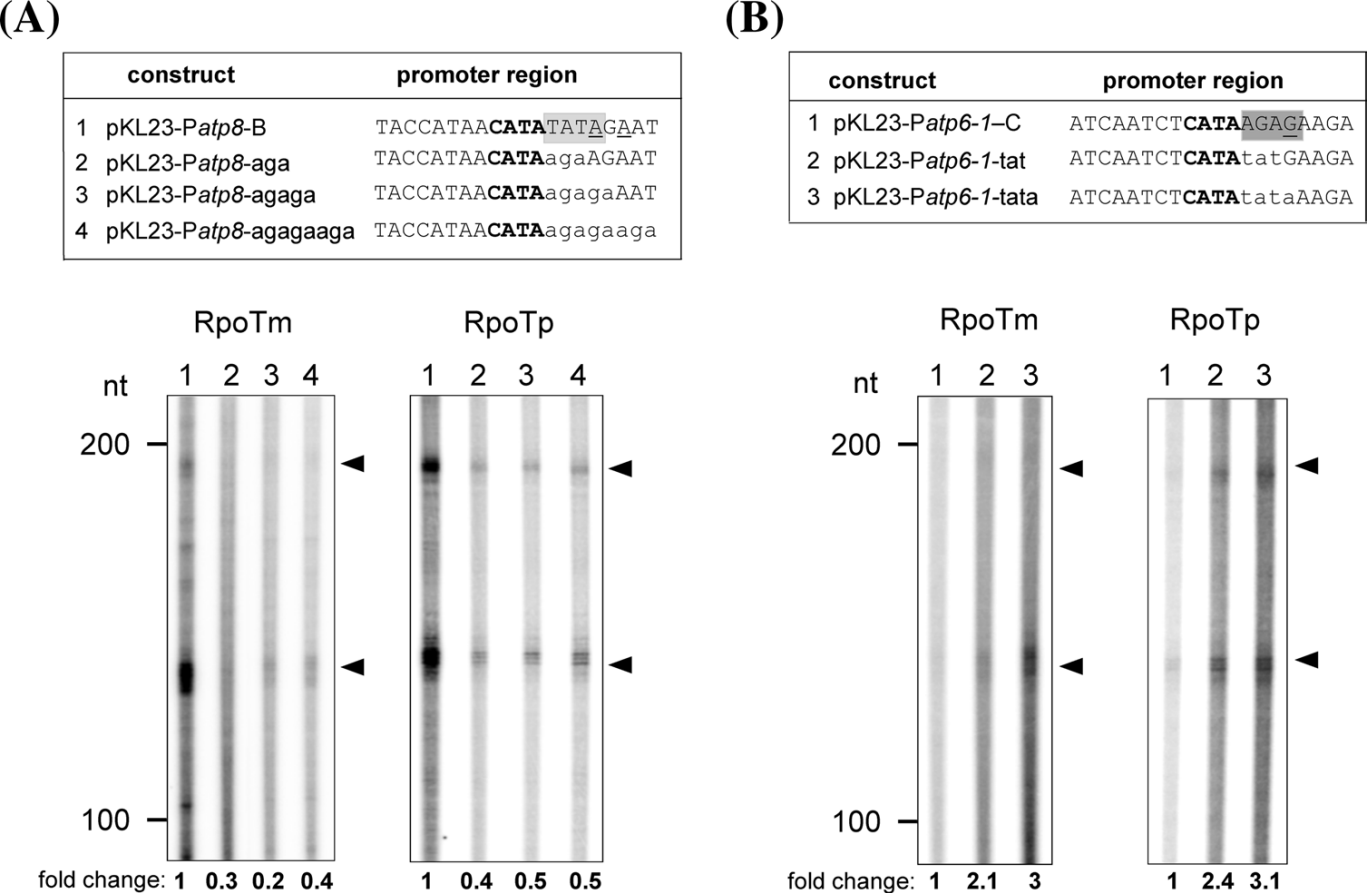
Mutational analysis of the TATA and AGAG promoter motifs. Recombinant RPOTm and RPOTp were assayed for promoter-specific transcription of the chimeric mitochondrial P*atp8*-228/226 (**a**) and P*atp6-1*-156 (**b**) promoters. *Upper panels* display wild-type and mutagenized promoter sequences which were inserted into pKL23 (compare Figs. 1a, 2a). The TATA and AGAG promoter motifs targeted by the mutagenesis are *highlighted in grey*. Nucleotides exchanged between the P*atp8*-228/226 and P*atp6-1*-156 promoters are *written in lower case* letters. *Lane numbers* in the *lower panels* correspond to promoter sequences shown *above*. Transcript analysis and *symbols* are as described in Fig. 1. Relative-fold changes for specific transcripts given below the autoradiogram were determined from the mean of two independent experiments

**Fig. 5:**
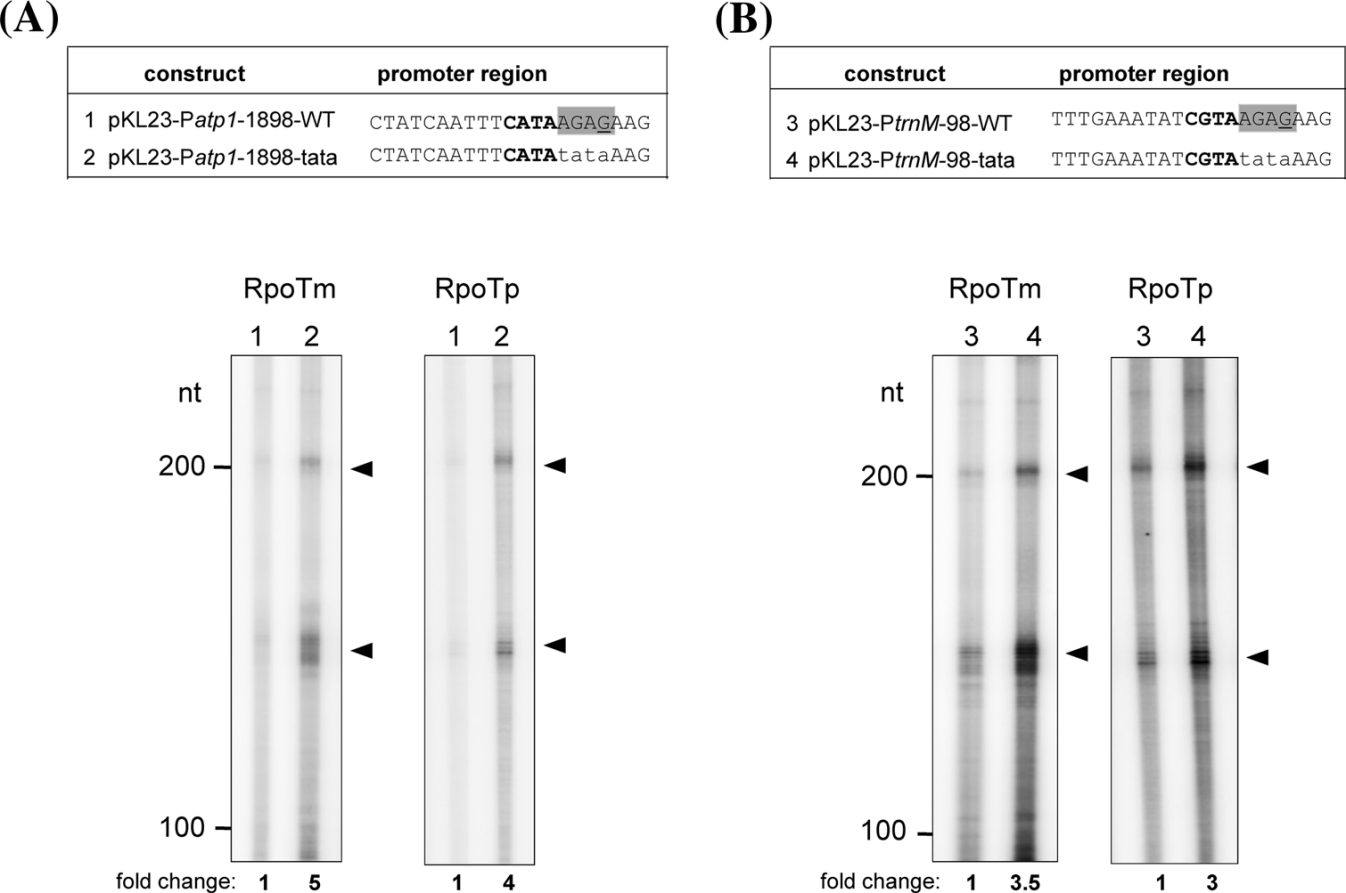
Mutational analysis of the AGAG motif in the mitochondrial P*atp1*-1898 and P*trnM*-98 promoters. Recombinant RPOTm and RPOTp were assayed for promoter-specific transcription of the mutagenized P*atp1*-1898 (**a**) and P*trnM*-98 (**b**) promoters. *Upper panels* display wild-type and mutagenized promoter sequences which were inserted into pKL23. The AGAG promoter motif targeted by the mutagenesis is *highlighted in grey*. Mutagenized nucleotides are written in *lower case* letters. *Lane numbers* in the *lower panels* correspond to promoter sequences shown *above*. Transcripts expected from initiation at the introduced promoter followed by termination at *hisa* and *thra* have a length of 159 and 228 nt, respectively. Transcript analysis and *symbols* are as described in Fig. 1. Relative-fold changes for specific transcripts given below the autoradiogram were determined from the mean of two (P*atp1-1898*) or three (P*trnM-98*) independent experiments

To elucidate the role of both motifs for promoter-specific transcription we investigated RNA synthesis from chimeric promoters. For this purpose, we selected the well-recognized P*atp8*-228/226 and the weakly transcribed P*atp6-1*-156 promoter, which both contain a CATA core sequence, but differ in the occurrence of the TATA or AGAG motifs (Fig. 4). RPOTm and RPOTp were assayed for transcription of chimeric promoters containing differently sized elements of the respective other promoter (Fig. 4a, b, upper panels). All variations of the P*atp8*-228/226 promoter considerably reduced the levels of specific RNAs synthesized by RPOTm and RPOTp to 10–50 % of transcript levels synthesized from the wild-type sequence (Fig. 4a). On the other hand, mutagenesis of the AGAG region in P*atp6-1*-156 stimulated transcription by both RNAPs approximately 2–3 fold (Fig. 4b). We additionally tested if changing the AGAG motif to TATA stimulates transcription also from further mitochondrial promoters. As shown in Fig. 5, indeed transcription from mutagenized P*atp1*-1898 (Fig. 5a) and P*trnM*-98 (Fig. 5b) promoters led to 3–5-fold higher transcript levels in comparison to the wild-type sequences.

### Altered promoter recognition by mutagenized RPOTmp

Specific promoter recognition by the T7 and mitochondrial yeast RNAP (Sc-RPO41) involves the formation of hydrogen bonds between amino acids of a C-terminal specificity loop with certain nucleotides of the promoter sequence (Cheetham et al. 1999; Nayak et al. 2009; Rong et al. 1998). Although this specificity loop is not conserved in organellar phage-type RNAPs, sequence comparisons by Nayak et al. (2009) revealed a variable 26 ± 3 amino acid sequence element at a position corresponding to the Sc-RPO41 and T7 RNAP promoter recognition loops. The authors therefore speculated that these elements may be involved in promoter recognition in most or all of the phage-type RNAPs.

**Fig. 6:**
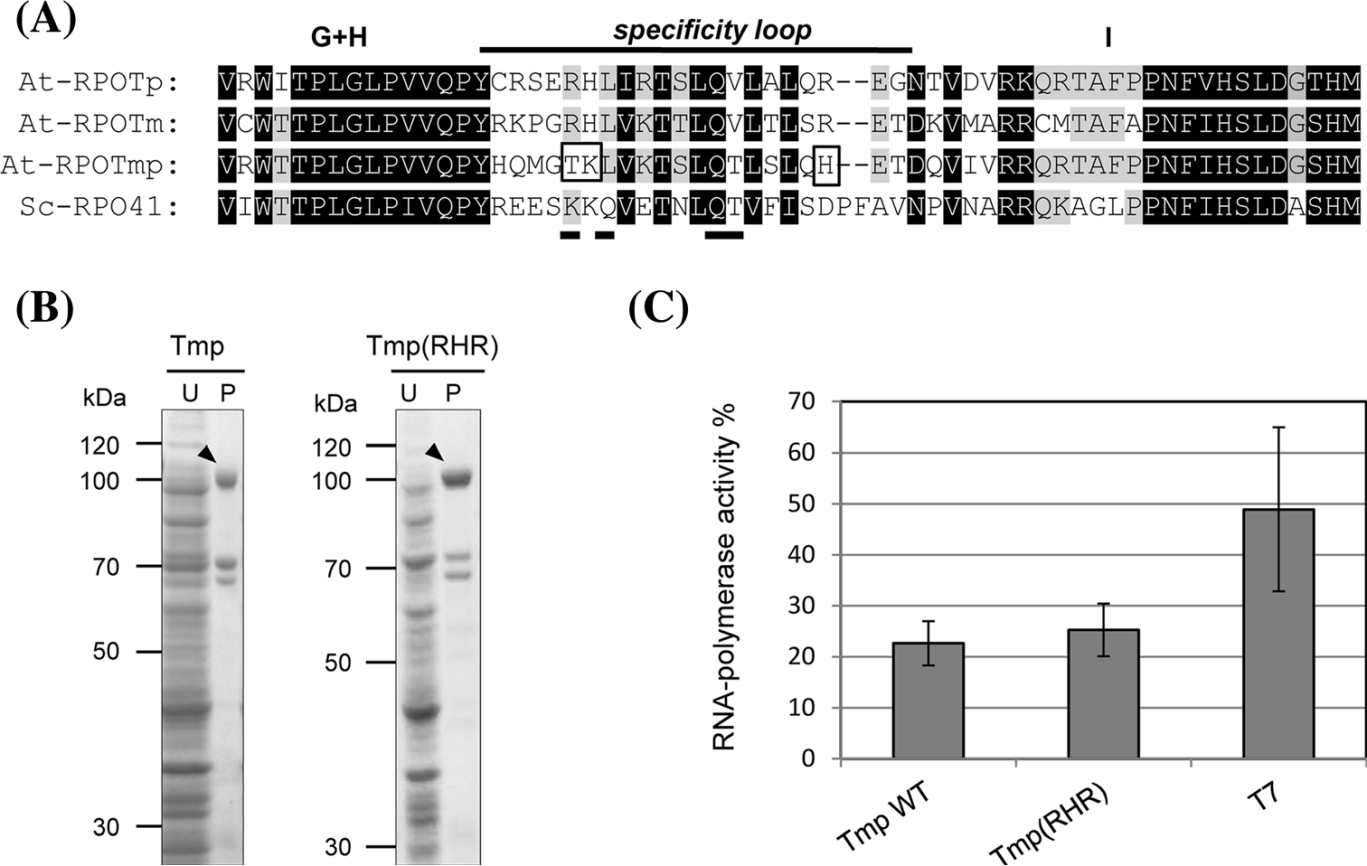
Mutagenesis of RPOTmp. **a** Amino acid sequence alignment of the putative specificity loop regions of the phage-type RNAPs from *A. thaliana* (At-RPOTmp, At-RPOTm, and At-RPOTp) and the mitochondrial phage-type RNAP from yeast (Sc-RPO41). The specificity loop region as well as the conserved G + H and I-blocks defined from sequence similarity of single subunit RNAP amino acid sequences by Cermakian et al. (1997) are displayed by GeneDoc software (http://external.informer.com/nrbsc.org/gfx%2Fgenedoc). *Shaded* positions are conserved in 75 % (*grey*) or 100 % (*black*) of aligned sequences. Amino acids that were exchanged in RPOTmp are boxed (RPOTmp: T884R, K885H, H898R). Amino acids of the yeast enzyme described to directly interact with bases of the promoter sequence are underlined (Nayak et al. 2009). **b** Coomassie-stained polyacrylamide gels demonstrating purification of recombinant RPOTmp wild-type (Tmp) and mutagenized proteins (Tmp (RHR)), respectively. Histidine-tagged proteins were expressed in *E. coli* by the pCOLD system, purified from bacterial extracts over Ni^2+^-agarose and separated by SDS–PAGE. Shown are total proteins of uninduced bacterial cultures (*U*) alongside the purified recombinant enzymes (*P*) which are designated by *arrow heads*. Sizes of the molecular mass marker are indicated in kilodaltons. The expected molecular weight for RPOTmp/RPOTmp (RHR) is 106 kDa. **c** Unspecific in vitro transcription by mutagenized RPOTmp. Incorporation of [α−^32^P]-UMP into transcripts synthesized in vitro by recombinant RPOTmp wild-type (*Tmp WT*) and mutagenized protein (*Tmp(RHR)*) from calf thymus DNA was determined using scintillation counting. 100 % transcriptional activity corresponds to a complete incorporation of ^32^P UTP into the RNA transcripts. As a positive control served the T7 RNA polymerase (*T7*). *Error bars* represent standard deviations from three independent experiments

The *Arabidopsis* enzymes RPOTm and RPOTp analysed in this study largely transcribe the same organellar promoters in vitro, while RPOTmp rarely shows specific and only weak promoter recognition (Bohne et al. 2007; Kühn et al. 2007). Therefore, the protein sequences within the putative specificity loop region of these enzymes were compared to obtain indications for the identity of amino acids, which, in contrast to RPOTmp, might enable RPOTm and RPOTp to specifically recognize the promoters (Fig. 6a). We focused mainly on positively charged amino acids, which are identical in RPOTm and RPOTp but different in RPOTmp and might confer binding of the oppositely charged DNA. We identified three such amino acids (R, H, R) within the specificity loop region of RPOTm and RPOTp (Fig. 6a). To test if these amino acids are involved in specific promoter recognition we exchanged these positions in RPOTmp (T, K, H) by mutagenesis of the recombinant enzyme to R, H, R. Soluble wild-type and mutagenized enzymes were purified from bacterial extracts (Fig. 6b) and assayed for transcription from a nonspecific DNA template to compare their general RNAP activities (Fig. 6c). As observed in our previous study (Kühn et al. 2007), the wild-type enzyme was capable of synthesizing RNA with high efficiency without initiation at specific promoter sequences. Unspecific RNAP activity of the mutagenized enzyme was comparable to that of the wild-type enzyme (Fig. 6c), thus indicating that the mutagenized RPOTmp (RHR) is still functional.

**Fig. 7:**
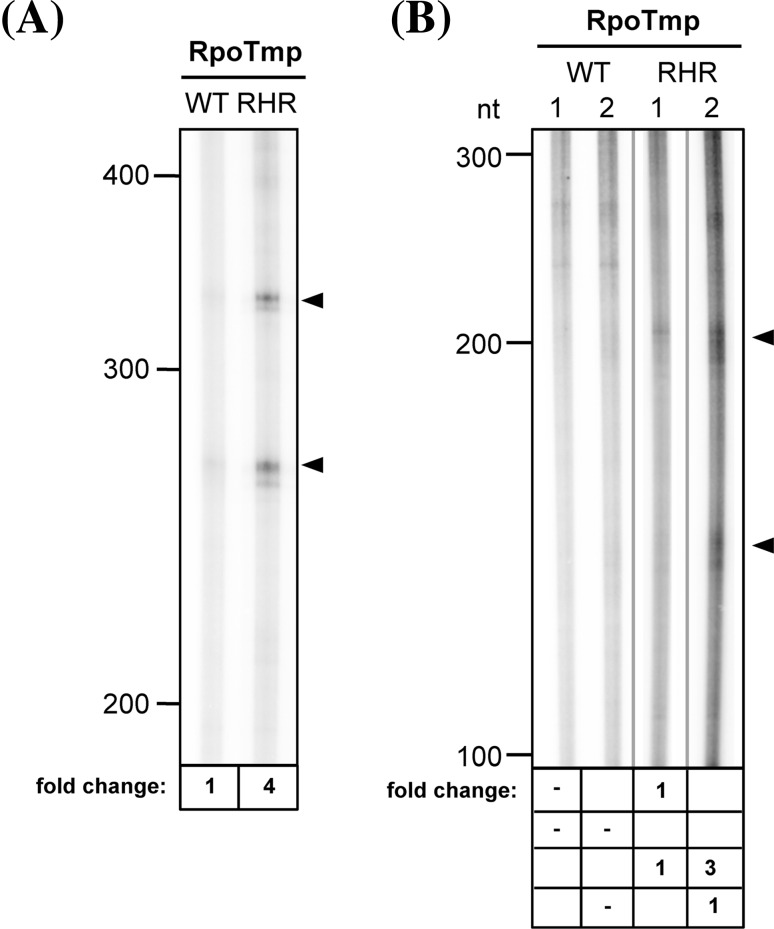
Specific in vitro transcription of P*atp8*-228/226 and P*trnM*-98 by mutagenized RPOTmp. **a** wild-type (*WT*) and mutagenized RPOTmp (*RHR*) were assayed for promoter-specific transcription from pKL23-P*atp8*-A (compare Fig. 1a). **b** Wild-type and mutagenized RPOTmp were assayed for promoter-specific transcription from pKL23-P*trnM*-98 (*lanes 1*) and pKL23-P*trnM*-98 tata (*lanes 2*) (compare Fig. 5b). Transcript analysis and *symbols* are as in Fig. 1. Relative-fold changes for specific transcripts given below the autoradiogram were determined from the mean of two independent experiments. Transcripts in (**b**) were separated on the same gel but not immediately adjacent to each other which is indicated by *grey vertical lines*

We next sought to investigate whether the mutagenized RNAP reveals an altered capability to recognize promoters (Fig. 7). Therefore, RPOTmp (RHR) was assayed for specific transcription from P*atp8*-228/226 (Fig. 7a). The RPOTmp wild-type enzyme hardly recognizes this promoter in vitro (Fig. 7a, left lane). Interestingly, mutagenesis of RPOTmp substantially increased the specific transcription from P*atp8*-228/226 (Fig. 7a, right lane).

In addition to the P*atp8*-228/226 promoter, we tested the mutagenized RPOTmp (RHR) for specific transcription from P*trnM*-98 as well as from the mutagenized P*trnM*-98-tata promoter, which enhanced transcription by RPOTm and RPOTp as described above (compare Fig. 5b). The RPOTmp (TKH) wild-type enzyme was neither able to specifically transcribe the P*trnM*-98 nor the P*trnM*-98-tata promoter (Fig. 7b). However, the exchange of only three amino acids in RPOTmp enabled the synthesis of specific RNAs from the wild-type and the mutagenized promoter sequences. The transcription by RPOTmp (RHR) was stimulated ~threefold by the insertion of the TATA element into the promoter region (Fig. 7) as seen for RPOTm and RPOTp (Fig. 5).

## Discussion

Like other dicotyledonous plants, the model plant *A. thaliana* has three nuclear-gene encoded phage-type RNAPs, which are involved in the transcription of genes in the two genome-bearing organelles, mitochondria and plastids: RPOTm, RPOTp, and RPOTmp. Intriguingly, RPOTm and RPOTp were found to initiate transcription correctly in in vitro assays without addition of further protein factors (Kühn et al. 2007) suggesting that the ability of promoter recognition by the single-subunit RNAP of phage T7 might have been retained during the evolution of phage-type RNAPs from bacteriophages up to higher plants. However, plastidial and mitochondrial promoter sequences used previously in in vitro assays with purified polymerases comprised 200–300 bp of DNA sequence around the in vivo initiation sites (Kühn et al. 2007), whereas in vitro analyses with transcriptionally competent mitochondrial and plastidial extracts (containing not only the RNAPs but also further transcription factors potentially needed for promoter recognition) identified promoter sequences of only 25 nucleotides or less comprising the transcription start site to be required for correct and efficient initiation of transcription (Canton et al. 1993; Caoile and Stern 1997; Dombrowski et al. 1999; Hanic-Joyce and Gray 1991; Rapp et al. 1993; Rapp and Stern 1992). Full transcriptional activity in vitro was achieved with the pea *atp9* promoter from the position −14 to +4, and the maize *atp1* promoter from nucleotides− 12 to +5 (Caoile and Stern 1997; Dombrowski et al. 1999; Rapp et al. 1993; Rapp and Stern 1992). Similarly, only a small DNA fragment from −15 to +5 relative to the transcription initiation site (+1) was found to be sufficient for correct transcription initiation in the case of the common Type-I NEP promoters of the chloroplast genome (P*atpB*-289; Kapoor and Sugiura 1999; P*rpoB*-345; Liere and Maliga 1999a, b; P*accD*-129; Xie and Allison 2002). Such in vitro assays with partially purified mitochondrial and chloroplast protein extracts are supposed to reflect the in vivo situation. We used therefore in the present study promoter sequences of similar size (−27 to +5, −26 to +6, −21 to +4, and −15 to +4 relative to the start point of transcription, respectively) but with different architectures. We observed efficient and precise initiation of transcription by the purified RPOTm and RPOTp polymerases without additional transcription factors in our in vitro assays as reported for transcriptionally active organellar protein extracts and for transcription in vivo. Thus, our data strongly suggest that the RNAPs recognize in vitro and in vivo identical promoter sequences.

Most analysed NEP promoters belong to the Type-I characterized by a conserved YRTA core motif located a few nucleotides upstream of the transcription start site. Plastidial promoters share this motif with the CRTA motif of many plant mitochondrial promoters. CRTA is part of a nonanucleotide sequence overlapping the initiation site in several promoter regions of dicot mitochondrial genomes (Binder et al. 1996). The YRTA/CRTA core motif showed a significant influence on the efficiency of in vitro transcription from the tobacco *rpoB* Type-Ia promoter in a plastidial in vitro transcription system (Liere and Maliga 1999a) and of several promoters using the transcriptional activity of mitochondrial protein extracts from monocots or dicots (Canton et al. 1993; Caoile and Stern 1997; Dombrowski et al. 1999; Hanic-Joyce and Gray 1991; Rapp et al. 1993; Rapp and Stern 1992). In good agreement with this data, our present investigation using promoters with mutagenized CRTA motif revealed its importance for transcriptional in vitro activity. In particular, changing the highly conserved TA resulted in a drastic reduction of promoter recognition by RPOTm and RPOTp, which confirms and extends a previous observation (Fig. 3a; Kühn et al. 2007). Vice versa, converting the original AGTA core sequence into cGTA, thereby creating a true CRTA motif, led to markedly enhanced initiation of transcription at the correct site, i.e. turns a weak promoter into a stronger one (Fig. 3b). Interestingly, also the substitution of AGTA to AGat, which is not described to be a promoter core sequence, led to increased transcription by RPOTm (Fig. 3b, lane 4). We can only speculate why this substitution increases the activity of this promoter. The mutagenesis might lead to the recognition of an alternative ATTA promoter core which is positioned a few nucleotides upstream of the created AGat sequence (P*atp6-2-436-AGat* TCTTGAATTA**AGat**TATAG) and has been described to be recognized by RPOTm in vitro (Kühn et al. 2007). The AGat motif on the other hand is often seen around the transcription start site of mitochondrial promoters (e.g. P*ccmC-1817*, P*ccmC-45*, P*nad2e1-413*; Kühn et al. 2009) and might therefore efficiently be used for transcription initiation in vitro and in vivo.

Previous analyses have shown that also nucleotides of the promoter region outside of the core motif affect the promoter strength in in vitro transcription assays with mitochondrial extracts. Mutagenesis of nucleotides of the pea *atp9* and the maize *atp1* promoter at positions −3, −2, and +1 drastically reduced the transcriptional activity in vitro (Caoile and Stern 1997; Dombrowski et al. 1999; Rapp et al. 1993; Rapp and Stern 1992). However, it has not been investigated yet whether certain sequence motifs in this region have a specific effect on promoter strength. The region just downstream of the CRTA box contains the sequence TATA or AGAG (first transcribed nucleotide +1 underlined) in many mitochondrial promoters and some NEP promoters of *Arabidopsis* (Kühn et al. 2005). Converting TATA into AGAG turned the strong P*atp8*-228/226 into a promoter, which was only weakly used by RPOTm and RPOTp. The relatively weak promoters P*atp6-1*-156, P*atp1*-1898 and P*trnM*-98, however, became more efficient when their original AGAG sequence was substituted by TATA (Figs. 4, 5). Our results therefore help to define elements within the promoter core and sequences around the initiation site which are likely to influence promoter usage also in vivo.

The plastid multisubunit bacterial-type RNAP needs a sigma factor for promoter recognition. Different sigma factors exist controlling promoter usage in response to developmental and environmental cues. Moreover, a few further transcription factors have been described which may affect the rate of transcription of plastid genes (Liere et al. 2011; Börner et al. 2015). In contrast, there is no evidence for regulation of the transcriptional activity of plant phage-type RNA polymerases by transcription factors (Liere et al. 2011) and no indication for differential promoter usage under changing environmental conditions or during development other than cases of RPOTmp-dependent transcription discussed below. Thus, setting the promoter strength by sequence motifs during evolution may be a major or even the only means to determine the level of primary transcripts in mitochondria and of RPOTp- and RPOTmp-dependent transcripts in plastids. In the following, we discuss therefore the questions as to whether RPOT polymerases may act also in vivo without auxiliary factors and as to wether the effects of sequence motifs on transcriptional activity observed in our in vitro assays could be the same as those in organello.

**Fig. 8:**
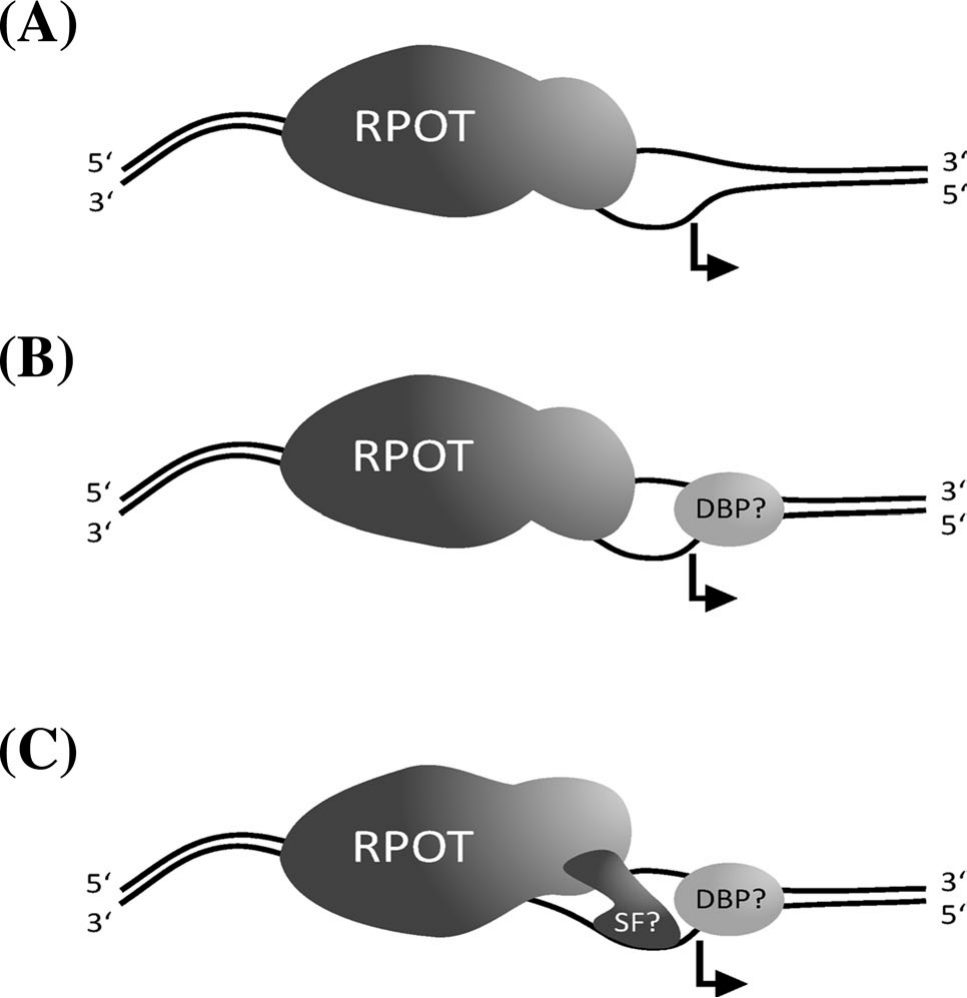
Organellar transcription initiation by phage-type RNAPs. Depending on the individual promoter there seem to be at least three modes of promoter recognition conceivable. While some promoters might be directly recognized by the intrinsic capacity of RPOTm/Tp after the promoter region has been opened by the RNAP alone (**a** with supercoiled DNA templates as a precondition) or with support of one or more hypothetical DNA-binding protein(s) (DBP) (**b**), others are likely to additionally require one or more associated and yet unidentified specificity factor(s) (*SF*) mediating the recognition of the promoter sequence and/or guiding the RNAP to the transcription start site (**c**)

In vitro promoter recognition was only observed with supercoiled DNA templates which facilitate opening of the DNA double helix as also described for the mitochondrial RNAP of yeast (Matsunaga and Jaehning 2004; Kühn et al. 2007). A smaller portion of the plant mitochondrial genomes may exist in form of circular DNA molecules of different size (Backert et al. 1997; Gualberto et al. 2014). Therefore, a low basic transcription could be performed by RPOTm from part of the promoters without auxiliary factors (Fig. 8a). Nevertheless, a larger portion of the mitochondrial genomes of angiosperms is proposed to exist in vivo as linear molecules of different size (Backert et al. 1997; Gualberto et al. 2014), thus would not be a suitable template for RPOTm. RPOTm will, according to our data, therefore need in organello support by transcription factors which assist in opening the double helix in the promoter region (Fig. 8b). Like in yeast mitochondria, it might be just one and the same factor for all promoters, and promoter strength would depend only on the sequence of the promoter recognized by the polymerase. In chloroplasts, a substantial part of the DNA is present in supercoiled conformation (Herrmann and Possingham 1980), which may allow RPOTp to transcribe genes from NEP promoters without the assistance of further factors (Fig. 8a). Yet, neither RPOTm nor RPOTp did recognize all promoters that were offered as template in our in vitro assays. Thus, transcription initiation by the RPOTs might be achieved by three different modes: (i) only by the catalytic subunit of the phage-type RNAP, which we observed only for part of the promoters and would be possible only for those promoters, which are located on supercoiled DNA molecules (Fig. 8a), (ii) by the catalytic subunit recognizing the promoter and supported in melting the double helix by one or more accessory factor(s) (DNA-binding protein(s) which might act like the yeast factor mtTFA; the necessary presence of this protein makes scenario (i) rather unlikely to occur; Fig. 8b), and (iii) by the catalytic subunit plus at least one factor that facilitates promoter recognition and melting or more factors with separate functions in recognition and melting (Fig. 8c). Therefore, the in organello promoter activity is likely be influenced by certain promoter elements which determine the efficiency by which they are recognized only by the phage-type RNAP. Additionally, transcription of at least some genes might be tuned by accessory specificity factors, which could be expressed e.g. only under certain growth conditions or at different developmental stages. At least modes (i) and (ii) (Fig. 8a, b) would imply that the effects of promoter motifs on RPOTs detected in our in vitro assays would be the same or very similar also in organello. Also the striking similarities between the results of our in vitro transcription assays and of the experiments with lysates of chloroplasts and mitochondria support the view that the effects of different promoter elements on transcription initiation observed in our experiments reflect truly the in organello situation. This could experimentally be tested by measuring transcriptional rates in transplastomic chloroplasts with correspondingly altered promoter sequences.

A specific case is RPOTmp, which in all our experiments barely recognized promoters in vitro. Nayak et al. (2009) identified a structural domain within the T7 and mitochondrial RNAPs which differs in sequence between the organisms but may nevertheless be critical for promoter recognition as experimentally shown for the T7 and yeast mitochondrial RNAP. They hypothesized that most or all phage RNAPs of the T3/T7 type and organellar RNAPs may recognize their promoters by this domain. Our data support this hypothesis. We created a mutant version of RPOTmp by adapting the amino acid sequence of the putative promoter recognition loop at three positions to the sequence of RPOTm and RPOTp. The obtained mutant version of RPOTmp was able to recognize promoters in a similar way as RPOTm and RPOTp (Figs. 5, 7) indicating that the supposed specificity loop in the three *Arabidopsis* RNAPs should indeed be involved in promoter recognition like in case of the T7 RNAP and the mitochondrial RNAP of yeast. Additionally, the interaction of at least one of the exchanged amino acids with the transcription stimulating TATA motif downstream of the promoter core is very likely as the insertion of this motif increased specific transcription by the mutagenized RPOTmp. However, even though the exchange of only three residues of the proposed specificity loop in RPOTmp was sufficient to enable promoter recognition, it is likely that further amino acids in RPOTm and RPOTp determine the identity of recognized nucleotides and efficiency of transcription. This is supported by the high variance of identified organellar promoter motifs as well as by our finding that RPOTm and RPOTp, which exhibit identical residues at these three positions, do not always recognize promoter sequences with the same efficiency (Fig. 1b, template 3; Fig. 3b, template 4; Kühn 2009).

Taken together, our study supports the hypothesis that the capability of promoter recognition has been conserved during the evolution of T3/T7 phage-type RNAPs from bacteriophages up to higher plants. RPOTmp appeared as the last of the phage-type RNAPs during the evolution of angiosperms (Weihe et al. 2012). According to our data, only a few mutations were needed to cause the putative loss of the ability for promoter recognition by RPOTmp. Since RPOTmp co-exists in plastids with RPOTp and in mitochondria with RPOTm, the need of specific accessory factor(s) for promoter recognition by RPOTmp as indicated by our results might have facilitated the evolution of specific functions for this enzyme in the transcription of plastid and mitochondrial genes (Courtois et al. 2007; Kühn et al. 2009). In this respect, the described gene- and not promoter-specific transcription of several mitochondrial genes by RPOTmp might guarantee a probably trans-factor dependent transcription of these genes despite of frequent rearrangements causing promoter substitutions within the mitochondrial DNA of higher plants (Unseld et al. 1997; Kubo et al. 1999; Handa 2003; Forner et al. 2008). If this holds true also for the function of RPOTmp in plastid transcription remains to be shown in the future. Moreover, beside several gene specific functions of RPOTmp, the enzyme might represent another nuclear encoded protein which, similar to the pentatricopeptide repeat (PPR) proteins, could be involved in “debugging of organellar genomes”. While the RNA-binding PPR proteins are proposed to compensate frequent mutations of the asexually reproducing organellar genomes on the transcript level (Maier et al. 2008), multiple promoters upstream of organellar genes as well as the possibility of less specific and promoter independent transcription by RPOTmp combined with its much higher activity compared to RPOTm and RPOTp might allow at least basic transcription levels even when promoters became non-functional due to mutations.

## Electronic supplementary material

Below is the link to the electronic supplementary material.

Supplementary Tables (DOC 64 KB)

## References

[CR1] Amunts A, Nelson N (2008). Functional organization of a plant Photosystem I: evolution of a highly efficient photochemical machine. Plant Physiol Biochem.

[CR2] Backert S, Nielsen BL, Börner T (1997). The mystery of the rings: structure and replication of mitochondrial genomes from higher plants. Trends Plant Sci.

[CR3] Binder S, Marchfelder A, Brennicke A (1996). Regulation of gene expression in plant mitochondria. Plant Mol Biol.

[CR4] Bohne A-V, Ruf S, Borner T, Bock R (2007). Faithful transcription initiation from a mitochondrial promoter in transgenic plastids. Nucleic Acids Res.

[CR5] Börner T, Aleynikova AY, Zubo YO, Kusnetsov VV (2015). Chloroplast RNA polymerases: role in chloroplast biogenesis. Biochim Biophys Acta.

[CR6] Canton FR, Garcia-Gutierrez A, Gallardo F, Vicente AD, Canovas FM (1993). Molecular characterization of a cDNA clone encoding glutamine synthetase from a gymnosperm, *Pinus sylvestris*. Plant Mol Biol.

[CR7] Caoile AG, Stern DB (1997). A conserved core element is functionally important for maize mitochondrial promoter activity in vitro. Nucleic Acids Res.

[CR8] Cermakian N, Ikeda TM, Miramontes P, Lang BF, Gray MW, Cedergren R (1997). On the evolution of the single-subunit RNA polymerases. J Mol Evol.

[CR9] Cheetham GM, Jeruzalmi D, Steitz TA (1999). Structural basis for initiation of transcription from an RNA polymerase-promoter complex. Nature.

[CR10] Courtois F, Merendino L, Demarsy E, Mache R, Lerbs-Mache S (2007). Phage-type RNA polymerase RPOTmp transcribes the *rrn* operon from the PC promoter at early developmental stages in *Arabidopsis*. Plant Physiol.

[CR11] Dombrowski S, Hoffmann M, Guha C, Binder S (1999). Continuous primary sequence requirements in the 18-nucleotide promoter of dicot plant mitochondria. J Biol Chem.

[CR12] Forner J, Hölzle A, Jonietz C, Thuss S, Schwarzländer M, Weber B, Meyer RC, Binder S (2008). Mitochondrial mRNA polymorphisms in different Arabidopsis accessions. Plant Physiol.

[CR13] Gualberto JM, Mileshina D, Wallet C, Niazi AK, Weber-Lotfi F, Dietrich A (2014). The plant mitochondrial genome: dynamics and maintenance. Biochimie.

[CR14] Handa H (2003). The complete nucleotide sequence and RNA editing content of the mitochondrial genome of rapeseed (*Brassica napus* L.): comparative analysis of the mitochondrial genomes of rapeseed and *Arabidopsis thaliana*. Nucleic Acids Res.

[CR15] Hanic-Joyce PJ, Gray MW (1991). Accurate transcription of a plant mitochondrial gene in vitro. Mol Cell Biol.

[CR16] Hansson A, Amann K, Zygadlo A, Meurer J, Scheller HV, Jensen PE (2007). Knock-out of the chloroplast-encoded PSI-J subunit of photosystem I in *Nicotiana tabacum*. FEBS J.

[CR17] Herrmann RG, Possingham JV, Reinert J (1980). Plastid DNA - the plastome. Results and Problems in Cell Differentiation, vol10. Chloroplasts.

[CR18] Jang SH, Jaehning JA (1991). The yeast mitochondrial RNA polymerase specificity factor, MTF1, is similar to bacterial sigma factors. J Biol Chem.

[CR19] Kapoor S, Sugiura M (1999). Identification of two essential sequence elements in the nonconsensus type II P*atpB*-290 plastid promoter by using plastid transcription extracts from cultured tobacco BY-2 cells. Plant Cell.

[CR20] Kühn K, Weihe A, Börner T (2005). Multiple promoters are a common feature of mitochondrial genes in *Arabidopsis*. Nucleic Acids Res.

[CR48] Kubo T, Nishizawa S, Mikami T (1999). Alterations in organization and transcription of the mitochondrial genome of cytoplasmic male sterile sugar beet (*Beta vulgaris* L.). Mol Gen Genet.

[CR21] Kühn K, Bohne A-V, Liere K, Weihe A, Börner T (2007). *Arabidopsis* phage-type RNA polymerases: accurate in vitro transcription of organellar genes. Plant Cell.

[CR22] Kühn K, Richter U, Meyer EH, Delannoy E, de Longevialle AF, O’Toole N, Börner T, Millar AH, Small ID, Whelan J (2009). Phage-type RNA polymerase RPOTmp performs gene-specific transcription in mitochondria of *Arabidopsis thaliana*. Plant Cell.

[CR23] Liere K, Maliga P (1999). In vitro characterization of the tobacco rpoB promoter reveals a core sequence motif conserved between phage-type plastid and plant mitochondrial promoters. EMBO J.

[CR24] Liere K, Maliga P, Argyroudi-Akoyunoglou JH, Senger H (1999). Novel in vitro transcription assay indicates that the *accD* NEP promoter is contained in a 19bp fragment. The chloroplast: from molecular biology to biotechnology.

[CR25] Liere K, Weihe A, Börner T (2011). The transcription machineries of plant mitochondria and chloroplasts: composition, function, and regulation. J Plant Physiol.

[CR26] Lisowsky T, Michaelis G (1988). A nuclear gene essential for mitochondrial replication suppresses a defect of mitochondrial transcription in *Saccharomyces cerevisiae*. Mol Gen Genet.

[CR27] Maier UG, Bozarth A, Funk HT, Zauner S, Rensing SA, Schmitz-Linneweber C, Börner T, Tillich M (2008). Complex chloroplast RNA metabolism: just debugging the genetic programme?. BMC Biol.

[CR28] Mangus DA, Jang SH, Jaehning JA (1994). Release of the yeast mitochondrial RNA polymerase specificity factor from transcription complexes. J Biol Chem.

[CR29] Matsunaga M, Jaehning JA (2004). Intrinsic promoter recognition by a “core” RNA polymerase. J Biol Chem.

[CR30] Nayak D, Guo Q, Sousa R (2009). A promoter recognition mechanism common to yeast mitochondrial and phage T7 RNA polymerases. J Biol Chem.

[CR31] Park AK, Kim H, Jin HJ (2009). Comprehensive phylogenetic analysis of evolutionarily conserved rRNA adenine dimethyltransferase suggests diverse bacterial contributions to the nucleus-encoded plastid proteome. Mol Phylogenet Evol.

[CR32] Pfalz J, Pfannschmidt T (2013). Essential nucleoid proteins in early chloroplast development. Trends Plant Sci.

[CR33] Rapp WD, Stern DB (1992). A conserved 11 nucleotide sequence contains an essential promoter element of the maize mitochondrial *atp1* gene. EMBO J.

[CR34] Rapp WD, Lupold DS, Mack S, Stern DB (1993). Architecture of the maize mitochondrial *atp1* promoter as determined by linker-scanning and point mutagenesis. Mol Cell Biol.

[CR35] Richter U, Kühn K, Okada S, Brennicke A, Weihe A, Börner T (2010). A mitochondrial rRNA dimethyladenosine methyltransferase in *Arabidopsis*. Plant J.

[CR36] Rong M, He B, McAllister WT, Durbin RK (1998). Promoter specificity determinants of T7 RNA polymerase. Proc Natl Acad Sci USA.

[CR37] Schinkel AH, Koerkamp MJ, Touw EP, Tabak HF (1987). Specificity factor of yeast mitochondrial RNA polymerase. Purification and interaction with core RNA polymerase. J Biol Chem.

[CR38] Schubot FD, Chen CJ, Rose JP, Dailey TA, Dailey HA, Wang BC (2001). Crystal structure of the transcription factor sc-mtTFB offers insights into mitochondrial transcription. Protein Sci.

[CR39] Shadel GS, Clayton DA (1995). A *Saccharomyces cerevisiae* mitochondrial transcription factor, sc-mtTFB, shares features with sigma factors but is functionally distinct. Mol Cell Biol.

[CR40] Sousa R, Mukherjee S (2003). T7 RNA polymerase. Prog Nucleic Acid Res Mol Biol.

[CR41] Swiatecka-Hagenbruch M, Liere K, Börner T (2007). High diversity of plastidial promoters in *Arabidopsis thaliana*. Mol Genet Genomics.

[CR42] Swiatecka-Hagenbruch M, Emanuel C, Hedtke B, Liere K, Börner T (2008). Impaired function of the phage-type RNA polymerase RpoTp in transcription of chloroplast genes is compensated by a second phage-type RNA polymerase. Nucleic Acids Res.

[CR43] Unseld M, Marienfeld JR, Brandt P, Brennicke A (1997). The mitochondrial genome of *Arabidopsis thaliana* contains 57 genes in 366924 nucleotides. Nat Genet.

[CR44] Weihe A, Liere K, Börner T, Bullerwell CE (2012). Transcription and transcription regulation in chloroplasts and mitochondria of higher plants. Organelle Genetics.

[CR45] Xie G, Allison LA (2002). Sequences upstream of the YRTA core region are essential for transcription of the tobacco *atpB* NEP promoter in chloroplasts in vivo. Curr Genet.

[CR46] Yagi Y, Shiina T (2014). Recent advances in the study of chloroplast gene expression and its evolution. Front. Plant Sci.

[CR47] Zhelyazkova P, Sharma CM, Förstner KU, Liere K, Vogel J, Börner T (2012). The primary transcriptome of barley chloroplasts: numerous noncoding RNAs and the dominating role of the plastid-encoded RNA polymerase. Plant Cell.

