# Supplementary material for: In vitro promoter recognition by the catalytic subunit of plant phage-type RNA polymerases
Source: Plant Mol Biol. 2016 Aug 6;92(3):357–69. doi: 10.1007/s11103-016-0518-z (PMC5040748; doi:10.1007/s11103-016-0518-z)
Supplement: Supplementary file 1 — Supplementary Tables (DOC 64 KB) [file 11103_2016_518_MOESM1_ESM.doc]

## Table S1 Primers pairs used for the generation of annealing products directly ligated into pKL23. Lowercase nucleotides correspond to sequences added in order to introduce *Sac*I /*EcoR*I restriction sites. Promoter core sequences are written in bold, mutated nucleotides are underlined.

| **Primer pair** | **Primer sequences (5’→3’)** | **Construct** | **NEP-promoters** |
| --- | --- | --- | --- |
| AtPrrn18 (-156,WT) F  AtPrrn18 (-156,WT) R | cATCGATTGATAGAATAATA**CGTA**TATAATCAGg  aattcCTGATTATATACGTATTATTCTATCAATCGATgagct | pKL23-P*rrn18*-B | P*rrn18*-156 |
| AthPatp8-2 (-233C,-232C) F  AthPatp8-2 (-233C,-232C) R | cGATCTTTTTCCATACCATAA**CACC**TATAGAATg  aattcATTCTATAGGTGTTATGGTATGGAAAAAGATCgagct | pKL23-P*atp8*-CAcc | P*atp8*-228/226 |
| AthPatp8-2 (-234T,-233G) F  AthPatp8-2 (-234T,-233G) R | cGATCTTTTTCCATACCATAA**CTGA**TATAGAATg  aattcATTCTATATCAGTTATGGTATGGAAAAAGATCgagct | pKL23-P*atp8*-CtgA |
| AthPatp8-2 (-235A,-234C) F  AthPatp8-2 (-235A,-234C) R | cGATCTTTTTCCATACCATAA**ACTA**TATAGAATg  aattcATTCTATATAGTTTATGGTATGGAAAAAGATCgagct | pKL23-P*atp8*-acTA |
| Ath-Patp8-2 (-228/-226) F  Ath-Patp8-2 (-228/-226) R | cGATCTTTTTCCATACCATAA**CATA**TATAGAATg  aattcATTCTATATATGTTATGGTATGGAAAAAGATCgagct | pKL23-P*atp8*-B |
| At-Patp8-2 (-228/-226) kurzF  At-Patp8-2 (-228/-226 ) kurzR | cTACCATAA**CATA**TATAGAAg  aattcTTCTATATATGTTATGGTAgagct | pKL23-P*atp8-*C |
| AtPatp8-2/Patp6-1-156 F  AtPatp8-2/Patp6-1-156 R | cGATCTTTTTCCATAC**CATA**ACATAagagaagag  aattcTCTTCTCTTATGTTATGGTATGGAAAAAGATCgagct | pKL23-P*atp8-*agagaaga |
| AtPatp8-2 agaga F  AtPatp8-2 agaga R | cGATCTTTTTCCATAC**CATA**ACATAAGAGAAATg  aattcATTTCTCTTATGTTATGGTATGGAAAAAGATCgagct | pKL23-P*atp8*-agaga |
| AtPatp8-2 (-228/-226) agaaF  AtPatp8-2 (-228/-226) agaaR | cGATCTTTTTCCATAC**CATA**ACATAAGAAGAATg  aattcATTCTTCTTATGTTATGGTATGGAAAAAGATCgagct | pKL23-P*atp8*-agaa |
| AtPatp8-2 (-228/-226) agagF  AtPatp8-2 (-228/-226) agagR | cGATCTTTTTCCATACCATAA**CATA**AGAGGAATg  aattcATTCCTCTTATGTTATGGTATGGAAAAAGATCgagct | pKL23-P*atp8*-agag |
| AtPatp6-1 (-200,WT) F  AtPatp6-1 (-200,WT) R | cAGTGCAGCAGCCAATAATA**CGTA**TATAAGAAGg  aattcCTTCTTATATACGTATTATTGGCTGCTGCACTgagct | pKL23-P*atp6-1-B* | P*atp6-1*-200 |
| AtPatp6-1 (-156,WT) F  AtPatp6-1 (-156,WT) R | cCGGGATCAAACTATCAATCT**CATA**AGAGAAGAg  aattcTCTTCTCTTATGAGATTGATAGTTTGATCCCGgagct | pKL23-P*atp6-1-C* | P*atp6-1*-156 |
| AtPatp6-1 (-156) tatg F  AtPatp6-1 (-156) tatg R | cCGGGATCAAACTATCAATCT**CATA**TATGAAGAg  aattcTCTTCATATATGAGATTGATAGTTTGATCCCGgagct | pKL23-P*atp6-1-*tat |
| AtPatp6-1 (-156) tata F  AtPatp6-1 (-156) tata R | cCGGGATCAAACTATCAATCT**CATA**TATAAAGAg  aattcTCTTTATATATGAGATTGATAGTTTGATCCCGgagct | pKL23-P*atp6-1-*tata |
| AtPatp6-2 (-436,WT) F  AtPatp6-2 (-436,WT) R | cTTGCTTTCATCTTGAATTA**AGTA**TATAGAAAAg  aattcTTTTCTATATACTTAATTCAAGATGAAAGCAAgagct | pKL23-P*atp6-2-*B | P*atp6-2*-436 |
| AtPatp6-2 (-436,ccTA) F  AtPatp6-2 (-436,ccTA) R | cTTGCTTTCATCTTGAATTA**CCTA**TATAGAAAAg  aattcTTTTCTATATAGGTAATTCAAGATGAAAGCAAgagct | pKL23-P*atp6-2-*ccTA |
| AtPatp6-2 (-436,AccA) F  AtPatp6-2 (-436,AccA) R | cTTGCTTTCATCTTGAATTA**ACCA**TATAGAAAAg  aattcTTTTCTATATGGTTAATTCAAGATGAAAGCAAgagct | pKL23-P*atp6-2-*AccA |
| AtPatp6-2 (-436,AGat) F  AtPatp6-2 (-436,AGat) R | cTTGCTTTCATCTTGAATTA**AGAt**TATAGAAAAg  aattcTTTTCTATAATCTTAATTCAAGATGAAAGCAAgagct | pKL23-P*atp6-2-*AGat |
| AtPatp6-2 (-436,cGTA) F  AtPatp6-2 (-436,cGTA) R | cTTGCTTTCATCTTGAATTA**CGTA**TATAGAAAAg  aattcTTTTCTATATACGTAATTCAAGATGAAAGCAAgagct | pKL23-P*atp6-2-*cGTA |
| AtPycf1 (-39,WT) F  AtPycf1 (-39,WT) R | cGGAAAGTTGCAAAATCAT**CATA**TAATAATCCAg  aattcTGGATTATTATATGATGATTTTGCAACTTTCCgagct | pKL23-*ycf1-B* | P*ycf1*-39 |
| AtPatp1 (-1898,WT) F  AtPatp1 (-1898,WT) R | cTCTCACAAACTATCAATTTCATAAGAGAAGAAAGg  aattcCTTTCTTCTCTTATGAAATTGATAGTTTGTGAGAgagct | pKL23-P*atp1*-1898-WT | P*atp1*-1898 |
| AtPatp1 (-1898) tata F  AtPatp1 (-1898) tata R | cTCTCACAAACTATCAATTTCATATATAAAGAAAGg  aattcCTTTCTTTATATATGAAATTGATAGTTTGTGAGAgagct | pKL23-P*atp1*-1898-tata |
| AtPtrnM (-98,WT) F  AtPtrnM (-98,WT) R | cCTTCTTTCTTTTGAAATATCGTAAGAGAAGAAGGg  aattcCCTTCTTCTCTTACGATATTTCAAAAGAAAGAAGgagct | pKL23-P*trnM*-98-WT | P*trnM*-98 |
| AtPtrnM (-98) tata F  AtPtrnM (-98) tata R | cCTTCTTTCTTTTGAAATATCGTATATAAAGAAGGg  aattcCCTTCTTTATATACGATATTTCAAAAGAAAGAAGgagct | pKL23-P*trnM*-98-tata |

### **Table S2** Primer pair used for PCR-amplification of organellar DNA sequences and subsequent construction of *in vitro* transcription templates. Lowercase nucleotides correspond to sequences added in order to introduce *Sac*I /*EcoR*I restriction sites.

| **Primer pair** | **Primer sequences (5’→3’)** | **Construct** | **NEP-promoters** |
| --- | --- | --- | --- |
| Patp8-228/226 fw  Patp8-228/226-157rev | cagcgagctcCCTGTACATACAAAGATCTAGGCAGC  cagcctgcagAACAAAAGCATGGGAGAAAACC | pKL23-*atp8*-A | P*atp8*-157  P*atp8*-228/226 |

### **Table S3** Primer pairs used for PCR-amplification and site directed mutagenesis of coding sequences of the RNA polymerase RPOTmp. Lowercase nucleotides correspond to sequences added in order to introduce restriction sites for cloning into the pCOLD vector. Underlined nucleotides represent introduced point mutations within the coding sequence of RPOTmp.

| **Primer pair** | **Primer sequences (5’→3’)** | **Construct** |
| --- | --- | --- |
| RpoTmp-pCOLD-Fw (*Sac*I)  RpoTmp-pCOLD-Rev (*Xba*I) | cagagctcAAGAGCGAGAGGTGCCTTAGT  cgtctagaTCGAGACCGAGGAGAGGGTTAG | pCold-His-RpoTmp |
| RpoTmp_mut_fw  RpoTmp_mut_rev | CAGACCCTATCGCTTCAGCGTGAAACTGATCAGGTC  GAGGGATGTCTTTACGAGGTGTCTTCCCATTTGGTG | pCold-His-RpoTmp (RHR) |
